# The impact of the first COVID-19 lockdown on weight management practices in UK adults: A self-regulation perspective

**DOI:** 10.1177/20551029231214058

**Published:** 2023-11-08

**Authors:** Denisa Genes, Fuschia M Sirois, Nicola J Buckland

**Affiliations:** 1 7315 University of Sheffield, UK; 2 3057 Durham University, UK

**Keywords:** COVID-19 lockdown, self-regulation, obesity, weight management, energy intake, physical activity

## Abstract

This study aimed to identify the impact of the first UK COVID-19 lockdown on individuals’ weight management attempts (WMA). A self-regulation theoretical framework was used to identify predictors of continuing with a WMA, and weight change during the lockdown. An online retrospective cross-sectional study was conducted after the first UK COVID-19 lockdown. The sample consisted of 166 UK adults (M:31.08, SD:12.15) that were trying to manage their weight before the lockdown started. The survey assessed changes in WMA and practices, and measured perceived stress, flexible/rigid restraint, uncontrolled eating, craving control, and self-compassion. Results showed that 56% of participants reported disruption to their WMA during the lockdown. Participants with lower levels of perceived stress and higher flexible restraint were more likely to continue their WMA. Flexible restraint was a significant predictor of weight change. Interventions that promote flexibility in weight management may be beneficial for at-risk individuals under lockdown conditions.

## Introduction

In response to the first UK COVID-19 lockdown, all face-to-face weight management services were suspended with some adapting to digital delivery and others ceasing entirely. There was also closures to services instrumental to physical activity and dietary intake, which are two main behaviours often targeted as part of weight management attempts (Brown et al., 2021; UK. Gov., 2020). A Public Health England report showed that 60.6% of individuals in Tier 2 weight management services, and 78.3% in Tier 3 stated that their appointments had been canceled or delayed (Ells et al., 2020; Moussa et al., 2021). Additionally, there have been widespread reports that the COVID-19 lockdown had a negative impact on multiple health behaviours in general samples (e.g. Dicken et al., 2022; Naughton et al., 2021). However, there is limited evidence on how the COVID-19 lockdown impacted individuals who were attempting to manage their weight. This evidence is important because a considerable proportion of individuals attempt to manage their weight. Prior to the COVID-19 lockdown, 42% of the population reported trying to lose weight and 23% reported trying to maintain weight (Santos et al., 2017). Weight management (WM) has health benefits (Magkos et al., 2016) and small weight gain in a short period can lead to permanent substantial weight gain over time (Schoeller, 2014). Therefore, it is important to assess the impact of the COVID-19 lockdown on individuals’ WMA (either with professional support or self-led) and their ability to self-regulate weight protective behaviors.

Self-regulation of health behaviors is necessary for WM. Self-regulation refers to the ability to activate, monitor, inhibit and adapt behavior in response to internal cues, environmental stimuli, and feedback from others to attain personally relevant goals (Moilanen, 2007). There were multiple aspects about the lockdown rules that likely challenged the ability of some individuals to self-regulate weight management behaviors, such as dietary intake. For instance, the lockdown resulted in increased stocking up on comfort foods (Bhutani and Cooper, 2020). Additionally, the necessity to stay indoors resulted in constant exposure to these foods which may have consequently cued thoughts and increased food cravings (Boswell and Kober, 2016) and food intake (Nicola et al., 2020). Furthermore, the COVID-19 pandemic led to increased levels of fear, stress, sadness, and guilt (Brooks et al., 2020). Emotional distress and cue exposure can lead to dietary lapses (Forman et al., 2017; Goldstein et al., 2018). Indeed, in samples not specifically engaged in WMA, there have been reports of increased energy intake to soothe negative emotions during COVID-19 (Cherikh et al., 2020). As such, the COVID-19 lockdown may have challenged self-regulation and increased the likelihood of lapses, and been detrimental to WMA.

However, the negative effects of COVID-19 on WMA likely varied across individuals. According to self-regulation theory, various individual characteristics can help people face adversity and continue their self-regulation attempts (Teixeira et al., 2015). Some of these characteristics include self-compassion and eating behaviour traits such as flexible restraint and craving control. Self-compassion refers to a kind and understanding attitude towards oneself when faced with pain or failure (Neff, 2003). Self-compassion helps with self-regulation by fostering goal setting, taking action, evaluation of behavior, and emotional regulation (Sirois et al., 2015). In the COVID-19 situation, the extent to which individuals engaged in self-compassion may have influenced the ability to regulate stress, manage temporary relapses and therefore adhered to a WMA.

In terms of dietary restraint, flexible restraint involves a balanced approach to eating, by engaging in behaviors such as compensating at a subsequent meal for previous overconsumption (Westenhoefer et al., 1999). Flexible restraint is linked with improved weight loss outcomes (Westenhoefer et al., 2013). Rigid restraint on the other hand is described as a strict dichotomous, all-or-nothing approach to eating and WM (Westenhoefer, 1991). Therefore, individuals with higher flexible restraint may have been better able to adapt their dietary intake, engage in less uncontrolled eating and adhere to their WMA during the COVID-19 lockdown.

Additionally, flexible restraint could be beneficial to WM by supporting effective management of food cravings experienced during the WMA (Meule et al., 2011). A food craving refers to the intense desire to eat a certain food (Weingarten and Elston, 1990). Higher levels of food cravings are associated with increased BMI and disordered eating (Hill, 2007; Taetzsch et al., 2020). A low ability to control cravings (low craving control) has also been identified as a strong predictor of increased energy intake during the COVID-19 lockdown (Buckland et al., 2021; Buckland and Kemps, 2021). As such, the ability to refrain from acting on food cravings (high craving control) may have supported WM during the COVID-19 lockdown (Smithson and Hill, 2017).

Multiple studies have investigated the impact of COVID-19 on health behaviors in general samples (Bakaloudi et al., 2022; Stockwell et al., 2021). There are also emerging studies and reports on the experience of people engaged in weight loss attempts. A brief report on a commercial WM program showed that participants found it difficult to manage their weight during the COVID-19 lockdown (EASO, 2020). Furthermore, there have been some reports on changes to WMA during COVID-19 (Brown et al., 2021; Pellegrini et al., 2021). However, it remains unclear which individuals are most likely to digress from WMAs. Although some people gained weight and decreased their engagement in weight-related behaviors (Robinson et al., 2020), some used this period as an opportunity to change their lifestyle and make it healthier (Allabadi et al., 2020; Deschasaux-Tanguy et al., 2020). Furthermore, people living with obesity that were taking part in a weight management program reported that restrictions both helped and hindered their weight loss attempt (Thomson et al., 2022). This suggests there were individual differences in response to the lockdown and that further research is necessary to investigate individual differences and identify predictors of the impact of COVID-19 lockdowns on WMA.

Therefore, this study aimed to identify the impact of the first UK COVID-19 lockdown on self-regulation of weight-related behaviors from the framework of self-regulation theory. Specifically, the study aimed to identify: (i) changes in WMA and strategies; (ii) characteristics of individuals that continued their WMA; (iii) predictors of WMA continuation and weight change. It was hypothesized that the COVID-19 lockdown would have an impact on WMA, with most individuals reporting disruptions to their WMA and strategies used. Additionally, it was expected that the impact of the lockdown on WMA would vary across individuals. Specifically, it was expected that higher levels of self-compassion, craving control, flexible restraint, and lower levels of rigid restraint, stress and uncontrolled eating would be related to continued WMA. Finally, it was expected that stress, self-compassion, craving control, flexible/rigid restraint and uncontrolled eating would significantly predict changes in WMA and percentage weight change in response to the COVID-19 lockdown.

## Methods

### Participants and procedure

Data was collected from an online retrospective cross-sectional survey (via Qualtrics, Provo, UT) conducted after the first UK COVID-19 lockdown (September 1^st^ – November 9^th,^ 2020). Participants were recruited through social media and volunteer lists. A sample of 346 participants was targeted, based on an estimated small effect size (*r* = .15) and power of 0.80 (Ellis, 2010). The final sample consisted of 166 adults (*M:* 31.1, *SD*: 12.2 years). Whilst this sample size fell short of the planned sample size, Ellis (2010) suggests that this is sufficient to detect a medium effect size correlation coefficient (*r* = .25). To be eligible, participants needed to report engagement in a WMA (either with professional support or self-led) when the lockdown started. Respondents with a current or history of eating disorders were excluded (responded “yes” to “Do you have a current or history of an eating disorder?”). After providing informed consent, participants were asked to complete demographic information (e.g., age, gender) and screening questions (dieting status, eating disorders). Participants were then asked to complete questions about changes in WM strategies, eating behavior and physical activity in response to the COVID-19 lockdown. Participants then completed measures about stress, cognitive restraint, self-compassion, and craving control in a randomized order. Participants were then asked about their dieting history, postcode [to indicate socioeconomic status (SES)], general health (Jylhä, 2009), and COVID-19 status (e.g. infected, high-risk group; of note, participants were not excluded based on current COVID-19 infections) and impact (e.g. on income, caring responsibilities). All data in this study was self-reported. At the end of the questionnaire, participants were debriefed and had the opportunity to enter a prize draw. Two attention check questions were included for quality control, and participants that answered both incorrectly were excluded from the analysis. The study protocol was pre-registered on OSF. The study was ethically approved by the University of Sheffield ethics committee. The survey took on average 27.5 ± 11 min to complete.

### Measures

#### Outcomes

##### Weight management attempt

Dieting status in response to the lockdown was measured with a single item “What happened to your weight management attempt in response to the COVID-19 lockdown?” with four response options: stopped, continued, temporarily stopped, or other.

##### Engagement in weight management strategies

Changes in WM strategies were measured by asking participants whether engagement in certain strategies had changed during the lockdown compared to before. The strategies used were selected from The Oxford Food and Activity Behaviors (OxFAB) taxonomy (Hartmann-Boyce et al., 2016). For this study only a selection of these strategies was assessed (Appendix SA). Engagement with each strategy was assessed using a 100-point scale (0 – extremely decreased, 100 – extremely increased) and included the option “not applicable”. Results were evaluated at the domain level and for strategies deemed essential for weight management.

##### Eating behavior

Changes in eating behavior during the COVID-19 lockdown were measured using three items adapted from previous COVID-19 work (Buckland et al., 2021). Participants were asked to indicate the extent to which their eating habits changed in response to the lockdown. The questions assessed changes to overall food intake, snacks, and meals. Participants first stated whether their food intake had changed, and then indicated the amount of change on a scale ranging from “0 = extremely decreased” to “100 = extremely increased”.

##### Physical activity

Changes to physical activity was measured using the single-item physical activity measure (Milton et al., 2011). Participants indicated the number of days they engaged in 30-min of moderate physical activity in a typical week, both before the lockdown, and during the lockdown. The Single item physical activity measure is a valid tool for measuring changes in physical activity (O’Halloran et al., 2020). Participants also reported general changes in the frequency and duration of physical activity in response to the lockdown (0 - extremely disagree to 100 - extremely agree) using questions generated for this study.

##### Weight change

The Dieting and Weight History Questionnaire (DWHQ) was used to assess weight changes (Witt et al., 2013). Participants were asked to report their weight before the first lockdown (weight close to 23^rd^ March 2020) and their current weight. Percentage weight change since the beginning of lockdown was computed by deducting current weight from weight before the lockdown. As such, a higher number represent weight gain.

#### Predictors (Cronbach’s α in Table S1)

##### Perceived stress

Perceived stress was measured using the Perceived Stress Scale (PSS) (Cohen et al., 1983). The PSS is a measure of general perceived stress and assesses the degree to which individuals find their lives unpredictable, uncontrollable, and overloading. The response scale ranged from 0 (never) to 4 (very often). Higher scores indicate higher levels of perceived stress (Cronbach’s α: .89).

##### Self-compassion

The Self-Compassion Scale was used to measure the main components of self-compassion as well as their negative counterparts: self-kindness/self-judgement, common humanity/isolation, mindfulness/over-identification (Neff, 2003). Responses to the items ranged from 1 (almost never) to 5 (almost always). Higher scores indicate higher levels of self-compassion. In line with previous evidence (Neff, 2003) in the current sample the scale demonstrated good internal consistency α = .93.

##### Flexible and rigid restraint

To measure flexible and rigid restraint, the Flexible and Rigid Control of Dietary Restraint was used (Westenhoefer et al., 1999). The questionnaire provides a score for both flexible and rigid restraint. Higher scores per scale, indicate greater flexible and rigid restraint (Flexible restraint Cronbach's α = .79; Rigid restraint Cronbach's α = .80.).

##### Uncontrolled eating

Uncontrolled eating was measured using the revised version of The Three-Factor Eating Questionnaire (TFEQ-R18) (Karlsson et al., 2000). Uncontrolled eating refers to the tendency to overeat, and food intake being out of control. Higher scores indicate higher levels of uncontrolled eating (Cronbach’s α: .90.).

##### Craving control

Craving control was assessed with the Control of Eating Questionnaire (COEQ) (Dalton et al., 2015). The scale consists of five items measuring the severity and control over food cravings that an individual experiences over the previous 7 days. Responses were assessed using a 100-point scale with higher scores indicating greater craving control (Cronbach’s α: .92.).

SES was measured to assess whether reported changes in WM practices varied according to SES (Clemmensen et al., 2020; Darmon and Drewnowski, 2008). Participants were asked to provide their postcode to determine Index of Multiple Deprivation (IMD) (Scottish Government, 2020; StatsWales, 2020; UK Government, 2019). The IMD ranks small geographical areas in the UK. Deciles are reported and scores ranged from “1 = most deprived” to “10 = least deprived”.

As data was collected retrospectively (from 1^st^ September to 9^th^ November 2020), the number of days between the start of the lockdown and survey completion was computed to be used as a covariate in the analyses where relevant.

### Statistical analysis

Reported height, weight, weight change and computed BMI were screened to check for values outside of expected ranges (height between <1.40 and >2.20 m, weight <40 and >200 kg, weight change >40 kg and BMI <15 kg/m^2^ and >60 kg/m^2^). Data points for incomplete surveys were retained. Averages were not computed to fill in missing data points. To compare demographic information (e.g., age, sex) of completers and non-completers, t-tests and chi-squared tests were conducted. Out of the 431 participants that expressed interest in taking part in the study (accessed the survey link) 79 did not consent to take part. A further 182 provided consent but dropped out before completing the survey. 19 were excluded due to an eating disorder and four were excluded for incorrectly answering both attention check questions or having a completion time of less than 10 min. Therefore, sample sizes vary for each variable reported.

ANOVAs were conducted to compare the characteristics of individuals that continued, temporarily stopped, or terminated their WMA. Associations between variables were explored using bivariate correlations (Pearson’s *r*, *r* < .3 = small, .3 - .5 = medium, *r* > .5 = large. (Cohen, 1992). Two regression models were run to identify predictors of continuing with the WMA and percentage (%) weight change. Self-compassion, flexible/rigid restraint, uncontrolled eating, stress, and craving control were entered into the model as predictors (hierarchical method). For weight change (%), SES and days passed since the start of the lockdown were entered in the first step, and all other predictors were entered at step 2. Mahalanobis, Cooks, and Leverage scores indicated that there were no outliers. There were no issues with multicollinearity as based on the variance inflation factor (VIF <10), and tolerance values (>0.2) (Tabachnick and Fidell, 2013). The criterion for significance was *p* < .05. For the between-subjects comparison, effect sizes are reported (Cohen’s *d*: small = 0.2, medium = 0.5 large = 0.8). Statistical analysis was carried out using IBM SPSS version 26.

## Results

### Sample

The final sample consisted of 166 adults (*M:* 31.1, *SD*: 12.2 years, range 18 to 72). Most of the sample was female, 70.5% (*n* = 117; male *n* = 45; other *n* = 4), from a white ethnic background (77.1%, *n* = 128), and had a high level of education (see Tables 1, S2, S3). Approximately 24% (*n* = 25) of the participants had a current BMI (*M*: 29.9, *SD*: 5.5) over 30, which is representative for this age group of UK individuals (NHS, 2019). Most participants (88%, *n* = 145) were following a self-led diet and (12%, *n* = 20) were part of a program. There were no significant differences between completers and non-completers for any sample characteristic variables measured (see Table S4).

**Table 1.** Participant characteristics.

Variable (total *n*)	*n* (%) or *M* (*SD)*
*Education level (166)*	
No formal qualifications	1 (0.6%)
1-4 GCSEs or equivalent qualifications	5 (3%)
5 GCSEs or equivalent qualifications	4 (2.4%)
Apprenticeship	1 (0.6%)
2 or more A-levels or equivalent qualifications	45 (27.1%)
Bachelor’s degree or equivalent	71 (42.8%)
Doctoral or higher education	37 (22.3%)
Other qualifications including foreign qualifications	2 (1.2%)
*Ethnic group*	
White	128 (77.1%)
Mixed or multiple ethnic groups	4 (2.4%)
Asian or Asian British	23 (13.9%)
Black, African, Caribbean, or Black British	6 (3.6%)
Prefer not to say	2 (1.2%)
Other	3 (1.8%)

### Changes in WMA and practices in response to COVID-19

Before the COVID-19 lockdown: 70.5% (*n* = 117) of participants were attempting to lose weight, 24.1% (*n* = 40) were attempting to maintain weight and 5.4% (*n* = 9) were trying to gain weight. In response to the lockdown, 39.8% (*n* = 66) of individuals reported continuing their WMA, 25.3% (*n* = 42) stopped and 30.7% (*n* = 51) stopped temporarily and then started again. Approximately 28% (*n* = 46) of participants reported losing weight, 35.2% (*n* = 58) reported gaining weight and 24.2% (*n* = 40) reported that their weight fluctuated during the COVID-19 lockdown.

#### Weight management strategies and weight change

**Figure 1.**
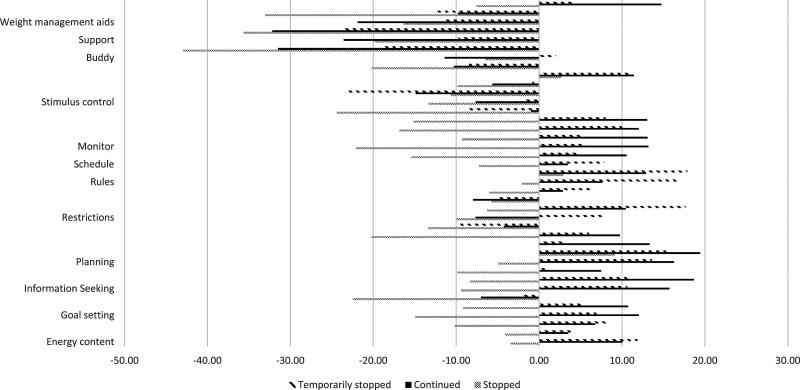
Difference in changes in weight management strategies between participant that stopped/continued or temporarily stopped and restarted their WMA. *Note:* Higher scores = increases.

Changes to specific WM strategies are shown in Figure 1. Approximately 42% of participants reported talking to a healthcare professional or having an online weight loss buddy. Participants also reported the highest decrease in engagement in these strategies due to the COVID-19 lockdown. Engagement in strategies related to planning meals, shopping and swapping foods increased overall. Decreased engagement in WM strategies was associated with weight gain since before the lockdown (Tables 3 and S5).

For the different strategy domains, most participants reported a decrease in strategies related to stimulus control (behaviours aimed at limiting exposure to food cues), use of WM aids, and seeking support. Changes to WM strategies significantly differed between participants that continued or had temporary disruptions and re-started to those that stopped their WMA. Participants that continued or had temporary disruptions to their WMA reported increased engagement in monitoring, information seeking, and setting rules compared to participants that stopped their WMA (Table S6).

**Figure 2.**
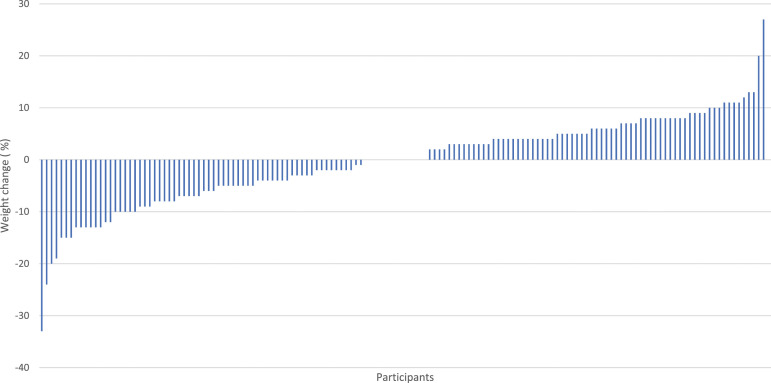
Individual variability in reported weight change (%) between pre- and post-COVID-19 first lockdown. *Note*. Negative values = weight loss and Positive values = weight gain.

Results showed that approximately 60% of participants reported some disruption to their WMA. Participants reported a mean weight change of −0.4 ± 8.4% (*M:* −0.8, *SD:* 7.59 kg). Around 40% of participants lost weight between the pre-COVID-19 lockdown and post-lockdown (Figure 2). As such, there was large individual variability in self-regulation ability, with some participants being more successful in self-regulating their weight during the lockdown than others.

### Characteristics of individuals that stopped, continued, or temporarily stopped their WMA

Table 2 shows the individual differences in eating behaviour traits of participants who continued, stopped, and temporarily stopped their WMA in response to the lockdown. Participants that continued their WMA reported significantly greater flexible restraint and craving control, and significantly less uncontrolled eating and perceived stress compared to participants that stopped their WMA. There were no significant differences between participants that stopped or continued their WMA in terms of self-compassion or rigid restraint. Participants that continued their WMA reported a significantly greater increase in physical activity and decrease in energy intake compared to participants that stopped their WMA (Table 3). These changes in energy intake (*r* = .45, *p* < .001) and physical activity (*r* = −.20, *p* < .05) were significantly correlated with reported percentage weight change. Specifically, participants that continued their WMA reported losing weight and those that stopped their WMA reported weight gain.

**Table 2.** Individual differences between participants that stopped (*n* = 42), continued (*n* = 66), or temporarily stopped (TD; *n* = 51) their weight management attempt after the COVID-19 lockdown.

Variable	Group	*M*(*SD*)	*F*	Cohen’s *d*
Weight change%	Stopped^a^	5.55 (6.85)	14.81**	1.40 (S/C)
	Continued^c^	−4.76 (7.85)		0.74 (C/T)
	TD^ac^	.94 (7.52)		0.64 (S/T)
Self-compassion	Stopped	2.68 (.73)	1.82	
	Continued	2.92 (.99)	*p* = .15	
	TD	2.55 (1.02)		
Rigid control	Stopped	39.61 (6.59)	1.52	
	Continued	39.32 (8.48)	*p* = .21	
	TD	40.36 (6.58)		
Flexible control	Stopped^a^	29.54 (5.92)	3.36*	0.47 (S/C)
	Continued^c^	32.09 (4.92)	*p* = .04	0.04 (C/T)
	TD^bd^	31.92 (4.88)		0.44 (S/T)
Uncontrolled eating	Stopped^a^	22.88 (5.26)	5.85**	0.58 (S/C)
	Continued^c^	19.82 (5.25)		0.54 (C/T)
	TD^ad^	22.92 (6.28)		0.01 (S/T)
Perceived stress	Stopped^a^	34.95 (5.01)	9.44**	0.80 (S/C)
	Continued^c^	30.54 (6.02)		0.67 (C/T)
	TD^ad^	34.98 (7.25)		0.01 (S/T)
Craving control	Stopped^a^	38.75 (25.92)	4.75*	0.56 (S/C)
	Continued^c^	52.95 (24.46)	*p* = .01	0.41 (C/T)
	TD^bd^	43.12 (23.8)		0.18 (S/T)

*p* < .001; * *p* < .05.

^a^Significant difference compared to participants that continued their WMA (*p* < .01).

^b^Non-significant difference compared to participants that continued their WMA.

^c^Significant difference compared to participants that stopped their WMA (*p* < .01).

^d^Non-significant difference compared to participants that stopped their WMA.

**Table 3.** Differences in eating behaviour and physical activity changes between participants that stopped (*n* = 42), continued (*n* = 66) or temporarily stopped (*n* = 51) their weight management attempt after the COVID-19 lockdown.

Variable	Group	*M(SD)*	*F*
Energy intake change	Stopped^a^	13.36 (17.14)	8.87**
	Continued^c^	−3.11 (13.64)	
	Temporary disruption^ad^	9.37 (22.02)	
Food amount	Stopped^a^	14.74 (21.67)	6.51**
	Continued^c^	−2.31 (16.75)	
	Temporary disruption^ad^	9.33 (23.97)	
Snack amount	Stopped^a^	20.69 (24.81)	6.74**
	Continued^c^	−0.88 (22.02)	
	Temporary disruption^bd^	10.60 (28.53)	
Meal change	Stopped^a^	4.64 (16.86)	5.27**
	Continued^c^	−6.32 (16.32)	
	Temporary disruption^ad^	6.66 (23.93)	
Physical activity (PA) change	Stopped^a^	−23.72 (23.94)	12.21**
	Continued^c^	4.68 (25.88)	
	Temporary disruption^ac^	−9.35 (22.41)	
PA day per week	Stopped^a^	−1.95 (1.87)	18.16**
	Continued^c^	1.79 (1.68)	
	Temporary disruption^ad^	−0.70 (2.28)	
PA time	Stopped^a^	−25.64 (28.41)	9.53**
	Continued^c^	5.83 (32.08)	
	Temporary disruption^ad^	−14.53 (33.29)	
Structured PA	Stopped^a^	−24.81 (30.51)	10.87**
	Continued^c^	11.03 (32.62)	
	Temporary disruption^bc^	−4.12 (33.23)	
Incidental PA	Stopped^a^	−20.71 (31.02)	3.47*
	Continued^c^	−2.82 (27.42)	
	Temporary disruption^bd^	−9.39 (32.30)	
BMI	Stopped^bd^	30.64 (3.75)	1.41
	Continued	28.91 (7.22)	
	Temporary disruption^bd^	31.28 (4.10)	

*Note.* ** *p* < .001; * *p* < .05.

^a^Significant difference compared to participants that continued their WMA (*p* < .01).

^b^Non-significant difference compared to participants that continued their WMA.

^c^Significant difference compared to participants that stopped their WMA (*p* < .01).

^d^Non-significant difference compared to participants that stopped their WMA.

### Predictors of WMA continuation

Multinomial logistic regression analysis was conducted to identify predictors of changes in WMA. The model included perceived stress, self-compassion, craving control, flexible/rigid control, and uncontrolled eating as predictors of the odds of participants stopping, continuing, or re-starting their WMA.

The final model was significant, *p* = .008, and perceived stress (*p* = .01) and flexible control (*p* = .04) were identified as significant discriminants of whether participants stopped or continued their WMA. Participants scoring high on flexible restraint (B = .14, *SE* = .06, *p* = .02) and low on perceived stress (B = -.12, *SE* = .05, *p* = .009) were more likely to continue their WMA during the COVID-19 lockdown. Participants scoring high on flexible restraint (B = .12, *SE* = .06, *p* = .04) were more likely to restart their WMA after disruption during the COVID-19 lockdown.

The model correctly predicted participants that continued their WMA 81.4% of the time. The model was less accurate at predicting participants that stopped (41.7%) or those that had temporary disruptions to their WMA (34.8%).

### Predictors of successful weight management

Regression analysis was conducted to identify predictors of weight change (%) during the COVID-19 lockdown. The regression model included perceived stress, self-compassion, craving control, flexible/rigid restraint, and uncontrolled eating as predictors and weight change as the outcome (Table 4). Number of days since the lockdown (*M:* 208; *SD:* 15 days) was added as a covariate in the regression model. The final regression model explained approximately 23% of the variance in weight change. Higher levels of flexible restraint significantly predicted greater weight loss. All other predictors and covariates were non-significant.

**Table 4.** Hierarchical linear regressions for individual characteristics regressed on weight change (%).

Outcome variable	B	*SE* B	β
Weight change %			
*Step 1*			
Constant	6.50	12.21	
SES	−0.09	0.28	−.03
Days	−0.03	0.06	−.05
*Step 2*			
Constant	37.35	15.17	
SES	−0.35	0.26	.002
Days	−0.09	0.05	−.11
Perceived stress	0.10	0.14	.08
Self-compassion	−0.36	0.92	−.04
Flexible restraint	−0.43	0.18	−.24*
Rigid restraint	−0.20	0.13	−.17
Uncontrolled eating	0.28	0.16	.20
Craving control	−0.08	0.04	−.21

*Note*. SES and days passed since the lockdown started were entered (enter method) as covariates in step 1, followed by all predictors in step 2 (hierarchical method).

For percentage weight change: *R*^*2*^ = .003, *p* = .83 for Step 1; *R*^*2*^ = 28, *p* < .001 for Step 2.

*p < .05.

B = unstandardized coefficient; B *SE* = unstandardized coefficient standard error; β = standardized coefficient.

SES = socioeconomic status.

## Discussion

In the current study, most participants reported disruption to their WMA in response to the COVID-19 lockdown. Individuals that stopped their WMA reported a decrease in physical activity and an increase in energy intake, which corresponded with an increase in weight. Individuals that continued their WMA scored higher in flexible restraint and craving control, and lower in uncontrolled eating and perceived stress compared to those who disengaged from their WMA. Flexible restraint and perceived stress were significant predictors of continuing with a WMA. Flexible restraint was also a significant predictor of weight change during the COVID-19 lockdown. Self-compassion did not have a significant direct effect on weight change.

Reported disruptions to WMA are in line with previous research from commercial WM programs (EASO, 2020) and people living with obesity (Brown et al., 2021) and show that the COVID-19 lockdown had a negative impact on about half of the individuals that were attempting to manage their weight. Disruptions to WMA coincided with reported changes in energy intake, physical activity, and WM strategies. These results extend current knowledge by providing further evidence on changes to specific WM strategies and provide evidence on disruptions to WMA in individuals from the general population who engaged in a self-led WMA (most previous research has focused only on those engaged in structured programs).

Responses to the COVID-19 lockdown varied largely across individuals, with some individuals reporting continued WMA and weight loss. Characteristics that helped individuals continue their self-regulation attempts included greater flexible restraint and craving control, and less uncontrolled eating and perceived stress. Perceived stress and flexible restraint were significant predictors of continuation with a WMA. Individuals with a more flexible approach to eating were also more successful in managing their weight. This is in line with previous research that suggested a positive relationship between flexible restraint and success in WM (Sairanen et al., 2014). Flexible restraint may be linked with WM as flexible approaches may result in greater acceptance and ability to adapt dietary intake in response to cravings and dietary lapses. For example, individuals scoring high in flexible restraint may initiate compensatory behaviors in response to lapses, rather than generating negative emotions that can lead to disengagement from a WMA. The current findings provide novel evidence which shows that flexible restraint is important for engagement in WMA attempts, especially during challenging times such as COVID-19.

Results showing the importance of perceived stress on WM are in line with self-regulation theory stating that negative affect is a barrier to self-regulation (Wagner and Heatherton, 2014). The mechanisms through which stress is affecting self-regulation include depleting cognitive resources (Hofmann et al., 2007), increasing emotional eating (Macht, 2008), and increasing preferences for immediate rewards over larger delayed ones (Tice and Bratslavsky, 2000). Therefore, individual characteristics that help deal with stress might be beneficial for WMAs.

Self-compassion is a quality that can facilitate the self-regulation of health behavior during challenging times by supporting adaptive emotion regulation (Sirois et al., 2015). In the current research, contrary to expectations, self-compassion was not a significant predictor of continuing with a WMA or weight change during the COVID-19 lockdown. Based on the current study, there is no evidence that taking a more compassionate attitude towards oneself directly benefits weight management under lockdown conditions. This might be due to the stressors of lockdown being above the threshold for which self-compassion may be beneficial. For example, evidence shows that reappraisal strategies (e.g., self-compassion) are less effective than suppression strategies for dealing with negative emotions (Diedrich et al., 2014; Sirois et al., 2019).

There are some limitations to this research. First, the data was collected retrospectively. To minimize the influence of this, time passed since the lockdown and survey completion was accounted for in the analysis. Second, the data collected is cross-sectional and self-reported. Research shows that individuals tend to underestimate dietary intake and overestimate physical activity (Dahle et al., 2021; Silsbury et al., 2015) and we have no information on how often or well the WM strategies measured were used. However, associations between reported energy intake and physical activity and weight change (Table S7) indicate that the measures used were sensitive to detect variability in responses as they aligned with expected associations (e.g., individuals that reported increased energy intake also reported gaining weight during the lockdown *r =* .45), suggesting validity in the measures used. Third, this sample of participants might not be representative of the general population given that it consists of primarily of highly educated white women. This might be the result of the recruitment methods used that were limited by the lockdown rules imposed by the COVID-19 lockdown rules at the time. However, the percentages of participants with a BMI over 30 kg/m^2^ is similar to the one reported in the general population. Finally, no pre-COVID-19 data were collected, therefore we have no baseline data to compare the current results to. An alternative explanation to the current results could be that participants responded to the WM questions on WM strategies and WMA based on the way their weight changed.

Nevertheless, the current study applied a theoretical framework to identify predictors of continuing with a WMA and weight change during a viral pandemic. This novel data provides evidence of important individual characteristics linked with the self-regulation of weight-related behaviors during challenging times. While some studies have reported on the individual characteristics of increased food intake during COVID-19 (Buckland et al., 2021; Robert et al., 2022), to our knowledge this is the first study to report on the individual characteristics associated with successful WM during COVID-19 in adults who were engaged in a WMA at the onset of the first COVID-19 lockdown. This is noteworthy because previous COVID-19 research is mainly derived from individuals not actively trying to manage their weight. Furthermore, the limited available evidence is from participants in structured WM programs (e.g. commercial or local authority commissioned) rather than self-led WMA (Ells et al., 2020; Pellegrini et al., 2021), yet most people who attempt WM adopt self-led approaches (Santos et al., 2017). The current research expands knowledge on the impact of COVID-19 lockdown on individuals following a self-led WMA. This is noteworthy as it captures a unique facet of the real-life experience of individuals dealing with overweight or obesity during a distinct historical period. Furthermore, it successfully captures the experiences of individuals with milder forms of obesity, an aspect often disregarded in existing literature.

This research also provides novel evidence on the importance of emotion regulation and a more flexible approach to eating behavior for the continuation of WMAs and weight change in the context of major disruptions to everyday life (COVID-19 lockdown). Emotional regulation and a flexible approach to eating are modifiable individual characteristics that can be trained (Rahimi-Ardabili et al., 2018; Sairanen et al., 2014). Additionally, individuals scoring low in flexible restraint can be targeted and provided with more support during risky periods. Given evidence that self-compassion interventions are effective for improving the self-regulation of health behaviors (Biber and Ellis, 2019), future research could also investigate the effects of promoting self-compassion for WM in individuals susceptible to WM lapses during stressful times. This evidence is important in developing interventions that will help individuals deal with risky periods such as the COVID-19 outbreaks and future lockdowns which are likely to happen (Xu and Li, 2020). As well as help better target support and guidance for more vulnerable people such as people living with overweight and obesity. This is noteworthy as this group is generally stigmatized and were identified as a risk group for COVID-19 and stigmatized during the pandemic (Farrell et al., 2022; Townsend et al., 2020).

## Conclusions

This study provides novel findings on the impact of the COVID-19 lockdown on weight management attempts, by using self-regulation theory to identify modifiable individual characteristics that predict the continuation and success of WMA in times of added stress. The current results indicate that perceived stress and flexible control of eating behavior are significant predictors of engagement and success in self-regulation of weight during the COVID-19 lockdown. These results have important implications for the development of future interventions for weight management.

## Supplemental Material

Supplemental Material - The impact of the first COVID-19 lockdown on weight management practices in UK adults: A self-regulation perspective Click here for additional data file. Supplemental Material for The impact of the first COVID-19 lockdown on weight management practices in UK adults: A self-regulation perspective by Denisa Genes, Fuschia M Sirois and Nicola J Buckland in Health Psychology Open.

## Data Availability

Data that support the findings for this study are available on OSF upon reasonable request https://osf.io/g39hn/.
