# Supplementary material for: The impact of the first COVID-19 lockdown on weight management practices in UK adults: A self-regulation perspective
Source: Health Psychol Open. 2023 Nov 8;10(2):20551029231214058. doi: 10.1177/20551029231214058 (PMC10637132; doi:10.1177/20551029231214058)
Supplement: Supplemental Material - The impact of the first COVID-19 lockdown on weight management practices in UK adults: A self-regulation perspective [file sj-pdf-1-hpo-10.1177_20551029231214058.pdf]

The impact of the COVID-19 lockdown on weight management practices in the UK: A self-regulation perspective

Supporting Information

Table S1: Cronbach's Alpha

**Scale**	**Cronbach's α**
Perceived Stress Scale	.89
The Self-Compassion Scale	.93
Flexible restraint	.79
Rigid restraint	.80
Uncontrolled eating	.90
Craving control	.92

Table S2: Sample health characteristics

Participant health characteristics.

Variable (total *n*)	*n* (%) or *M* ± *SD* (95% CI)
WMA before COVID-19 lockdown	
To gain weight	9 (5.4%)
Lose weight	117 (70.5%)
Avoid gaining weight	40 (24.1%)
WMA response to COVID-19 lockdown	
Continued WMA	66 (39.8%)
Stopped WMA	42 (25.3%)
Temporarily stopped WMA	51 (30.7%)
Other	7 (4.2%)
Health	
Pregnant	2 (1.2%)
Breastfeeding	1 (.6%)
Smoking	12 (7.2%)
Diabetes	3 (1.8%)
Heart disease	1 (.6%)
Under or overactive thyroid	10 (6 %)
Other health condition	20 (12 %)
No health condition	123 (74.1%)
Current health rating	
Excellent	14 (8.4%)
Very good	50 (30.1%)
Good	67 (40.4%)
Fair	29 (17.5%)
Poor	6 (3.6%)
Own weight classification	
Underweight	3 (1.8%)
Healthy weight	76 (45.8%)
Overweight	64 (38.6%)
Obese	23 (13.9%)

WMA = Weight management attempt

Table S3: Sample COVID-19 characteristics

Participant COVID-19 characteristics.

Variable (total *n*)	*n* (%) or *M* ± *SD* (95% CI)
COVID-19 lockdown status	
Self-isolated	20 (12%)
Went outside for essentials only	128 (77.1%)
Key worker	16 (9.6%)
Went to social gatherings	1 (.6%)
COVID-19 Status	
Contracted, confirmed by test	6 (3.6%)
Contracted, self-diagnosed	2 (1.2%)
Possibly contracted	23 (13.9%)
Not contracted, confirmed by test	29 (17.6%)
Don’t think so	105 (63.6%)
Living situation	
Alone	26 (15.7%)
Partner/spouse	80 (48.2%)
Children	29 (17.5%)
Friend(s)	6 (3.6%)
Parent(s)	51 (30.7%)
Siblings	29 (17.5%)
Grandparent(s)	3 (1.8%)
Housemate(s)	17 (10.2%)
Other	5 (3%)
COVID-19 employment status (164)	
Key worker	16 (9.8%)
Work from home	62 (37.8%)
Unable to work but is paid	9 (5.5%)
Did not work during first lockdown	7 (4.3%)
Not working, not being paid	6 (3.7%)
Made redundant	1 (.6%)
Not applicable	38 (23.2%)
Other	17 (10.4%)
Prefer not to say	8 (4.9%)

Table S4: Drop-out analysis

Participant characteristics.		
Variable	*n* (%) or *M* ± *SD* (95% CI)	
	Completers (171)	Drop-outs (182)
Age	30.88 (12.05)	29.06 (11.14)
Gender		
Male	45 (27.1%)	42 (23.1%)
Female	117 (70.5%)	130 (71.4%)
Other	4 (2.4%)	10 (5.5%)
Education level (166)		
No formal qualifications	1 (.6%)	3 (1.9%)
1-4 GCSEs or equivalent qualifications.	5 (3%)	3 (1.9%)
5 GCSEs or equivalent qualifications.	4 (2.4%)	4 (2.5%)
Apprenticeship.	1 (.6%)	
2 or more A-levels or equivalent qualifications.	45 (27.1%)	63 (39.1%)
Bachelors degree or equivalent.	71 (42.8%)	54 (33.5%)
Doctoral or higher education.	37 (22.3%)	32 (19.9%)
Other qualifications including foreign qualifications.	2 (1.2%)	2 (1.2%)
Ethnic group		
White	128 (77.1%))	121 (75.2%)
Mixed or multiple ethnic groups	4 (2.4 %)	8 (5%)
Asian or Asian British	23 (13.9%)	22 (13.7%)
Black, African, Caribbean, or Black British	6 (3.6%)	5 (3.1%)
Prefer not to say	2 (1.2%)	2 (1.2%)
Other	3 (1.8%)	3 (1.9%)

Table S5: Correlations between weight management strategies and Weight change %

Strategy	Weight change %	
	r	
Make up for overeating at one meal, by eating less at another	-.17	
Adjust the amount of food eaten based on physical activity	-.36	**
Make up for being sedentary one day by exercising more the next day	-.10	
Have a set goal of how much to eat each day	-.39	**
Have a set goal of how much physical activity to do each day	-.13	
Have a set goal of how much weight you want to lose each week	-.22	*
Make food choices based on the nutritional information on the food labels	-.28	**
Look up the nutrition information and/or calorie content of foods	-.29	**
Look up information on how many calories you burn doing physical activity	-.29	**
Plan meals in advance	-.23	**
Plan food shopping in advance (e.g. use a shopping list)	-.13	
Walk or cycle instead of driving or taking the bus	-.03	
Follow an exercise plan/routine	-.30	**
Do more chores at home/in the garden to get more exercise and lose weight	-.12	
Skip meals as a way to lose weight	-.15	
Avoid eating certain foods	-.35	**
Avoid specific shops or aisles in the supermarket	-.10	
Control portion size by putting a certain amount of food on a plate or drink in a glass	-.33	**
Drink water or a low-calorie drink or eat low-calorie food to limit the amount eaten during meals	-.29	**
Swap one type of food or drink for another that is better for the diet (e.g. lower fat or lower sugar versions of the same foods)	-.25	**
Plan meal times to help with weight loss plans	-.29	**
Schedule physical activity each week	-.17	
Keep track of the calorie and/or nutritional content of the foods eaten	-.40	**
Check the portion sizes	-.36	**
Keep track of physical activity	-.12	
Keep track of weight by weighing regularly	-.29	**
Measure waist (or other parts of the body)	-.16	
Use smaller plates, bowls or glasses when eating to help with portion control	-.24	*
Buy food pre-packaged in individual portions	-.05	
Buy smaller amounts of certain foods to help with eating less	-.15	
Don't buy or keep at home things that don’t fit with the diet	-.31	**
Do something to prompt exercise (e.g. lay out exercise clothes the night before)	-.13	
Try to lose weight alongside a friend/family member/partner	.18	
Have an online weight loss buddy	-.11	
Sought help to tackle feeling stressed, down, or anxious to avoid breaking the diet	-.03	

Talked to a healthcare professional about weight management (e.g. doctor, nurse, dietitian, physiotherapist, psychologist)	-.03
Use meal replacements (e.g. shakes, diet bars, etc.)	-.05
Use the gym	-.08
Exercise at home using own equipment or DVDs	-.16

**p<.01, *p<.05

Table S6: Mean (SD) differences in changes in weight management strategies (WMS) domains by weight management attempt (WMA) status (stopped (S), continued (C) or temporary disruption (T))

WMS domain	Stopped	Continued	Temporary disruption	S/T	S/C	C/T
Information seeking	-9.28 (30.36)	13.50 (22.30)	7.65 (26.33)	T(65)=-2.42**	t(78)=3.79***	t(93)=1.17
Rules	-0.63 (33.05)	9.50 (22.98)	16.94 (21.98)	T(76)=-2.80**	t(82)=1.66	t(88)=-1.56
Monitoring	-14.52 (26.42)	9.88 (20.95)	-0.19 (19.78)	T(52)=-2.28**	t(61)=4.04***	t(69)=2.06*
Support	-31.58 (23.14)	-32.38 (19.06)	-19.90 (25.29)	T(39)=-1.54	t(48)=-0.14	t(35)=-1.91

***p<.001
**p<.01
*p<.05

Table S7: Correlation Matrix

	1	2	3	4	5	6	7	8	9
**1. Weight Change%**									
**2. Energy intake change**	.45**								
**3. Physical activity change**	-.20*	-.24**							
**4. Craving control**	-.34**	-.40**	.11						
**5. Rigid control**	-.13	-.04	.01	-.10					
**6. Flexible control**	-.26**	-.15	.04	.14	.62**				
**7. Uncontrolled eating**	.30**	.39**	-.09	-.63**	.23**	-.09			
**8. Self-compassion**	-.16	-.21**	.10	.32**	-.25**	-.07	-.37**		
**9. Perceived stress**	.22**	.24**	-.18*	-.47**	.30**	.10	.45**	-.57**	

**p<.001

*p<.05

Appendix SA: The Oxford Food and Activity Behaviours (OxFAB) taxonomy and questionnaire (Hartmann-Boyce, Aveyard, Koshiaris, & Jebb, 2016).

The following questions are about changes in weight management strategies since the COVID 19 lockdown. When answering the questions, please consider your TYPICAL weight management practices before the lockdown and compare these to your TYPICAL weight management practices since the lockdown.

To answer the questions, please indicate on each scale the point that best represents your response to the question.

Each scale ranges from the extreme of '0 = I so this a lot less', '50 = I do this the same amount' to '100 = I do this a lot more').

To what extent do you use the following strategies to manage your weight.

Response scale

☐Does not apply at all

0	100
I do this a lot less	a lot more

1. Make up for overeating at one meal, by eating less at another.
2. Adjust the amount of food eaten based on physical activity.
3. Make up for being sedentary one day by exercising more the next day.
4. Have a set goal of how much to eat each day.
5. Have a set goal of how much physical activity to do each day.
6. Have a set goal of how much weight you want to lose each week.
7. Make food choices based on the nutritional information on the food labels.
8. Look up the nutrition information and/or calorie content of foods.
9. Look up information on how many calories you burn doing physical activity.
10. Plan meals in advance.
11. Plan food shopping in advance (e.g. use a shopping list).
12. Walk or cycle instead of driving or taking the bus.
13. Follow an exercise plan/routine.
14. Do more chores at home/in the garden to get more exercise and lose weight.
15. Skip meals as a way to lose weight.
16. Avoid eating certain foods.
17. Avoid specific shops or aisles in the supermarket.
18. Control portion size by putting a certain amount of food on a plate or drink in a glass.
19. Drink water or a low-calorie drink or eat low-calorie food to limit the amount eaten during meals.
20. Swap one type of food or drink for another that is better for the diet (e.g. lower fat or lower sugar versions of the same foods).
21. Plan meal times to help with weight loss plans.
22. Schedule physical activity each week.
23. Keep track of the calorie and/or nutritional content of the foods eaten.

24. Check the portion sizes.
25. Keep track of physical activity.
26. Keep track of weight by weighing regularly.
27. Measure waist (or other parts of the body).
28. Use smaller plates, bowls or glasses when eating to help with portion control.
29. Buy food pre-packaged in individual portions.
30. Buy smaller amounts of certain foods to help with eating less.
31. Don't buy or keep at home things that don’t fit with the diet.
32. Do something to prompt exercise (e.g. lay out exercise clothes the night before).
33. Try to lose weight alongside a friend/family member/partner.
34. Have an online weight loss buddy.
35. Sought help to tackle feeling stressed, down, or anxious to avoid breaking the diet.
36. Talked to a healthcare professional about weight management (e.g. doctor, nurse, dietitian, physiotherapist, psychologist).
37. Use meal replacements (e.g. shakes, diet bars, etc.).
38. Use the gym.
39. Exercise at home using own equipment or DVDs.
40. Please state any other strategies that you use to manage your weight.
41. Please use the comments box below to detail your experience of how the lockdown has affected your weight management attempt, this might include changes to the types and amounts of food you have eaten, physical activity or weight monitoring. If there are no changes, please type ‘no changes’ and move on to the next page.
